# Determining the prevalence and severity of cancer cachexia in advanced non-small cell lung cancer and its relationship with chemotherapy outcomes

**DOI:** 10.1007/s00520-019-05259-1

**Published:** 2020-01-08

**Authors:** Rhys White, C. Elizabeth Weekes, Robert Grant, Christine Baldwin, Hafez Ahmed

**Affiliations:** 1 Nutrition and Dietetics Department, Guy’s and St Thomas’ NHS Foundation Trust, Great Maze Pond, London, SE1 9RT UK; 2 Faculty of Health, Social Care and Education, St George’s, University of London, Cranmer Terrace, London, SW17 0RE UK; 3 Nutrition and Dietetics, Kings College London, Franklin-Wilkins Building, Waterloo, London, SE1 9NH UK

**Keywords:** Cancer, NSCLC, Cachexia, Classification, Chemotherapy

## Abstract

**Purpose:**

Cancer cachexia (CC) is a syndrome characterised by an ongoing loss of skeletal muscle mass associated with reduced tolerance to treatment. This study explored the prevalence and severity of CC in advanced non-small cell lung cancer (NSCLC) patients and determined its relationship with chemotherapy outcomes.

**Methods:**

CC was classified into a four-stage model: no cachexia, pre-cachexia (PC), cachexia and refractory cachexia (RC) with categorisation determined from biochemical and body composition and performance assessment. Associations between the stage of cachexia and chemotherapy outcomes including radiological response, the number of chemotherapy cycles completed and the number of cycles delayed or dose reduced were explored.

**Results:**

Twenty-four patients were included with 4 (18%) classified as having no cachexia, 4 (18%) PC, 3 (14%) cachexia (13.6%), and 11 (50%) RC. No association was observed between the stage of cachexia and the radiological response to chemotherapy number of cycles delayed or the number of cycle’s dose reduced; however, there was an association with the number of cycles completed (*p* = 0.030). An association between C-reactive protein (CRP) and the number of chemotherapy cycles completed (*p* = 0.044) and the number of dose reductions (*p* = 0.044) was also identified.

**Conclusions:**

Limited conclusions can be drawn given the small sample size. However, the majority of patients presented with some degree of cachexia at diagnosis. A relationship was identified between the increasing severity of cachexia and a lower number of chemotherapy cycles completed, as well as between CRP and the number of chemotherapy cycles completed and the number of dose reductions required, and therefore warrants further exploration in larger studies.

## Introduction

Cancer cachexia (CC) is a multifactorial syndrome characterised by an ongoing loss of skeletal muscle mass, with or without loss of fat mass that cannot be fully reversed by conventional nutritional support, which leads to progressive functional impairment [1]. It is associated with reduced quality of life; tolerance to treatment modalities, such as chemotherapy; and survival [2]. Due to its complex multifactorial nature, a standardised definition and diagnostic criteria have been difficult to establish which has hindered clinical trials and the management of this condition [3]. However, an international consensus group agreed a definition and classification framework that described cachexia as a continuum with three stages of clinical relevance that might be seen in one patient: pre-cachexia (PC), cachexia and refractory cachexia (RC) [1]. The framework recommends specific criteria for classification of each stage in terms of weight loss, body mass index (BMI) and the presence of sarcopenia. Other parameters thought to play a role in cachexia, such as anorexia and systemic inflammation, are discussed, but these parameters require further exploration to determine whether they should form part of the classification criteria.

Vigano et al. [4] applied the framework to a population which consisted of metastatic or recurrent non-small cell lung cancer (NSCLC) and gastrointestinal (GI) cancer patients. The results showed that for selected outcomes, including quality of life, hospital stay, chemotherapy dose reductions and survival, there was no difference between the PC and cachexia groups. However, these two groups were significantly different from those with no cachexia and RC with median survival in the no cachexia, cachexia and RC groups of 66.4, 39.4 and 11.1 weeks respectively (*p* < 0.001).

Other studies have investigated the impact of cachexia on clinical outcomes demonstrating poorer survival [5–8], reduced quality of life and greater burden of symptoms [5] although only one of these studies investigated a NSCLC population alone, with most including mixed cancer populations.

Two of the key features of CC are skeletal muscle wasting and systemic inflammation [1]. Skeletal muscle wasting, or sarcopenia, has been associated with reduced survival, reduced disease response and treatment-related toxicities in a wide range of cancers [9–12], independent of the presence of cachexia. C-reactive protein (CRP) has been extensively explored in advanced cancer patients as a proxy for inflammation with elevated levels associated with reduced performance status, survival [13] and poor nutritional status [14].

Many previous cachexia studies have included a number of tumour sites, which introduces some heterogeneity in terms of the population studied as we do not know if the metabolic changes that result from CC vary across different tumours and locations. The majority of different classification models lack validation and practical application to a clinical setting including a lack of criteria for what are thought to be important parameters such as inflammation and anorexia. There is also a limited understanding of the impact of some of these individual parameters on treatment outcomes.

The aims of this study were to explore the prevalence and severity of CC in a single tumour population of advanced NSCLC patients and to determine its relationship with chemotherapy outcomes.

## Materials and methods

### Study population

Newly diagnosed stage III (locally advanced disease) and stage IV (metastatic disease) NSCLC patients from a single cancer centre in England and > 18 years old were recruited for the study prior to commencing any anti-cancer treatment. Patients who had cancers at other sites or were already receiving cancer therapy were excluded.

### Planned recruitment rate

The intention was to approach all patients meeting the inclusion criteria throughout the data collection period, and therefore, no sampling was required, preventing any issues related to selection bias. Approximately 15 new NSCLC patients who start first line chemotherapy were seen at this study site each month. If all patients were approached for inclusion, assuming an 80% recruitment rate and a data collection period of 5 months, it would result in a total of 60 patients recruited. A high recruitment rate of 80% was assumed as little data were required from patients in addition to that already collected as part of their standard care.

### Clinical data

Relevant clinical characteristics (age, gender, diagnosis, cancer stage, co-morbidities and current medications) were recorded from electronic patient records.

### Assessing cancer cachexia

The presence and severity of CC was classified according to work by Vigano et al. [4] and is shown in Table 1 and included the measures detailed below.

**Table 1:** Cancer cachexia classification [4]

0 = no cachexia	
A	CRP > 10 or Alb < 32 g/dL or WBC > 11,000 or HgB < 120 g/L (men) 110 g/L (women)
B	Anorexia (ESAS appetite score ≥ 4/10) or decreased food intake (PG-SGA box 2 > 1)
C	≤ 5% WL over 6 months
Stage criteria	A alone, B alone or C alone or none of the above
1 = pre-cachexia	
A	CRP > 10 or Alb < 32 g/dL or WBC > 11,000 or HgB < 120 g/L (men) 110 g/L (women)
B	Anorexia (ESAS appetite score ≥ 4/10) or decreased food intake (PG-SGA box 2 > 1)
C	≤ 5% WL over 6 months
Stage criteria	A + B or A + C or B + C or A + B + C
2a = cachexia	
A	CRP > 10 or Alb < 32 g/dL or WBC > 11,000 or HgB < 120 g/L (men) 110 g/L (women)
B	Anorexia (ESAS appetite score ≥ 4/10) or decreased food intake (PG-SGA box 2 > 1)
D	> 5% WL over 6 months
E	PG-SGA box 4 ≤ 2 or hand-grip percentile ≥ 50
Stage cachexia	A + D + E or B + D + E or A + B + D + E
2b = cachexia caused by low BMI or sarcopenia	
A	CRP > 10 or Alb < 32 g/dL or WBC > 11,000 or HgB < 120 g/L (men) 110 g/L (women)
B	Anorexia (ESAS appetite score ≥ 4/10) or decreased food intake (PG-SGA box 2 > 1)
E	PG-SGA box 4 ≤ 2 or hand-grip percentile ≥ 50
F	BMI < 20 + WL > 2% or sarcopenia + WL > 2%, male sarcopenia: ASMI < 7.26 kg/m^2^, female sarcopenia: ASMI < 5.45 kg/m^2^
Stage criteria	A + E + F or B + E + F or A + B + E + F
3 = refractory cachexia	
A	CRP > 10 or Alb < 32 g/dL or WBC > 11,000 or HgB < 120 g/L (men) 110 g/L (women)
B	Anorexia (ESAS appetite score ≥ 4/10) or decreased food intake (PG-SGA box 2 > 1)
D	> 5% WL over 6 months
G	PG-SGA box 4 > 2 or hand-grip percentile < 50
Stage criteria	A + D + G or B + D + G or A + B + D + G

*Alb* albumin, *ASMI* appendicular skeletal muscle index, *BMI* body mass index, *CRP* C-reactive protein, *ESAS* Edmonton Symptom Assessment System, *HgB* haemoglobin, *PG-SGA* Patient-Generated Subjective Global Assessment, *WBC* white blood cell, *WL* weight loss

#### Physical performance/function

The World Health Organization (WHO) performance status [14] was recorded as part of the routine medical assessment, and the data was extracted from the patients’ medical record. Muscle strength was measured using hand grip in accordance with methodology recommended by the American Society of Hand Therapists [15] using a DynEx™ hand grip dynamometer (MD Systems Inc., Ohio, USA) in position 1. Results were categorised according to published centiles [16]. This was the only physical measurement required outside of the patient’s routine care.

#### Biochemical and haematological measurements

CRP (mg/L), white blood cell count (WBC) (10^9^/L), serum albumin (g/L) and blood haemoglobin (g/L) were recorded from routine blood results reported in the patients’ electronic medical record.

#### Anthropometric measurements

Weight in light clothing (kg) and height without shoes on a stadiometer (m) were recorded by clinic staff as part of routine care and recorded in the electronic medical record. BMI was calculated and categorised according to WHO criteria [17]. Patients were asked to report any unintentional weight loss experienced in the last 6 months and this was calculated as the percentage of weight lost.

#### Body composition and presence of sarcopenia

Muscle and fat masses were assessed from diagnostic computed tomography (CT) scans, and no CT scans outside of routine care were requested. Previous studies have validated a single slice abdominal image of skeletal and fat mass at the lumbar vertebral landmark, L3 with total body skeletal muscle and adipose tissue volumes [18]. Abdominal CT scans with a 5-mm slice thickness were used. Two consecutive images extending from the L3 in the inferior direction were assessed. Cross-sectional area (cm^2^) was calculated for each tissue on a workstation with automated specific tissue demarcation performed using the following HU thresholds: − 29 to + 150 (skeletal muscle excluding visceral organs), − 190 to − 30 (subcutaneous and intramuscular adipose tissue) and − 150 to − 50 (visceral adipose tissue). The mean values of the two scans were calculated and normalised for stature giving L3 skeletal muscle index in centimetre square per metre square (cm^2^/m^2^). Calculation of appendicular skeletal muscle index (ASMI) was used to determine whether patients were sarcopenic. Sarcopenia is defined as ASMI < 7.26 kg/m^2^ for males and < 5.45 kg/m^2^ for females [19]. CT analysis at L3 has been strongly related to ASMI with the above cutoff values corresponding to cutoffs for lumbar skeletal muscle index of 55.4cm^2^/m^2^ for males and < 38.9 cm^2^/m^2^ for females [20]. It is these values that were therefore used to define sarcopenia in this study.

#### Appetite and nutritional intake

Two short assessment tools were completed with the patients. Appetite was assessed using the Edmonton Symptom Assessment Scale (ESAS) where appetite is scored between 0 and 10 along a continuum where 0 equals no loss of appetite and 10 equals worst possible lack of appetite [21]. Dietary intake was assessed using box 2 of the Patient-Generated Subjective Global Assessment as per other cachexia classification studies [22].

### Chemotherapy outcomes

Data was collected from the patients’ medical records. Radiological response to chemotherapy was based on RECIST criteria version 1.1 [23]. Secondary outcomes included the number of chemotherapy cycles completed, the number of cycles delayed and the number of dose reductions.

### Statistical analysis

All data analysis was performed using the SPSS software (IBM, version 24). Categorical variables are presented as percentages with 95% confidence intervals (CIs) and continuous variables as means and standard deviations (SD) if normally distributed. Medians and interquartile ranges (IQRs) are presented where variables were not normally distributed. Spearman’s rank correlation or Kendall’s tau-B correlation as well as Fisher’s exact test were performed to explore relationships between selected variables. All results were deemed statistically significant when *p* ≤ 0.05, but significant findings between *p* = 0.01 and *p* = 0.05 should be treated as tentative, pending another analysis with more data.

### Ethics

This study was approved by NRES Committee London-Central and by the Faculty of Health, Social Care and Education at St George’s University, London. Informed written consent was obtained from all participants. Confidentiality, anonymity and other relevant ethical issues have been observed.

## Results

### Recruitment and data collection

A total of 24 patients were recruited to the study. Twenty-seven patients were approached to take part with only 3 declining demonstrating an 89% recruitment success exceeding that predicted (80%). However, the overall number recruited was considerably less than the 60 patients predicted. Full data sets were not achieved for 12 (50%) of patients as 5 patients (21%) did not have a CT scan available (either due to a scan not being performed or being of too poor quality to assess) and 5 patients (21%) missing laboratory results, mainly CRP.

### Demographic and baseline nutritional measures

The demographic and clinical data for all patients are shown in Table 2 with the nutritional measures and assessments shown in Table 3. The majority were males (*n* = 17, 71%), with a mean age of 65 years, and 17 (71%) had stage IV disease. Mean BMI was 23.4 kg/m^2^ with 4 patients (17%) having a BMI < 18.5 kg/m^2^ indicating the presence of malnutrition. Nine patients (38%) had lost > 10% of their body weight prior to recruitment, and 12 (50%) were classified as sarcopenic.

**Table 2:** Demographic and clinical characteristics

Variable	*n* (%)	Median (SD)
Age	24 (100)	64.8 (± 13.8)
Gender		
Male	17 (71)	
Female	7 (29)	
NSCLC sub-type		
Adenocarcinoma	2 (8)	
Squamous cell	18 (75)	
Non-differentiated	4 (17)	
Cancer stage		
Stage III (locally advanced)	7 (29)	
Stage IV (metastasised)	17 (71)	
Adrenal	4	
Brain	2	
Bone	7	
Liver	2	
Lung	8	
Chemotherapy received		
Yes	13 (54)	
No	10 (42)

**Table 3:** Cachexia measures and assessments

Variable	*n* (%)	Median (IQR)
Weight (kg)	24	66.7 kg (± 14.1)*
Height (m)	24	1.69 m (± 0.09)*
BMI (kg/m^2^)	24	23.4 (± 4.7)*
< 18.5	4 (17)	
18.5–24.9	12 (50)	
25–29.9	6 (25)	
≥ 30	2 (8)	
Weight loss in previous 6 months (%)	23	6.7 (0.5–17.0)
< 5%	9 (38)	
5–10%	5 (21)	
> 10%	9 (38)	
Skeletal muscle area L3 (cm^2^)	19	121.7 (103.3–154.7)
Total adipose tissue area L3 (cm^2^)	19	289.1 (104.0–402.6)
Skeletal muscle index L3 (cm^2^/m^2^)	19	42.9 (40.2–50.4)
Adipose tissue index L3 (cm^2^/m^2^)	19	101.6 (33.2–157.3)
Sarcopenia		
Yes	12 (50)	
No	7 (29)	
Hand grip strength (kg)	24	20.2 (15.7–26.8)
ESAS appetite score	24	4.5 (0.3–6.8)
Patient-Generated Subjective Global Assessment		
0	4 (17)	
1	9 (38)	
2	7 (29)	
3	4 (17)	
CRP (mg/L)	19	43.0 (5.0–82.0)
Albumin (mg/L)	21	39.0 (34.5–41.5)
Haemoglobin (g/L)	21	124.0 (107.5–143.5)
White cell count (× 10 × 9/L)	22	11.3 (8.6–16.8)
Performance status		
0	12 (50)	
1	2 (8)	
2	6 (25)	
3	4 (17)	

Median and IQR are reported except where the asterisk symbol indicates mean and SD

### Cachexia classification

Cachexia could be classified in 22/24 (92%) patients. Due to the small numbers of cases in each category and because of missing data, the PC and cachexia stages were collapsed to form one cachexia category, and results are shown in Table 4. Patients were classified with no cachexia (*n* = 4, 18%), PC (*n* = 4, 18%), cachexia (*n* = 3, 14%) (or combined PC and cachexia (*n* = 7, 32%)) and RC (*n* = 11, 50%).

**Table 4:** Prevalence and stage of cachexia

Cachexia stage	*n* (%)	95% CI of percentage
No cachexia	4 (18)	4.6–36.4
Combined PC + cachexia	7 (32)	13.6–50.0
Pre-cachexia	4 (18)	4.6–36.46
Cachexia	3 (14)	0.0–27.3
Refractory cachexia	11 (50)	27.3–72.7

### Stage of cachexia and chemotherapy outcomes

Only 13/24 (52%) patients recruited went on to receive chemotherapy with the rest either having no treatment, palliative radiotherapy only or biological therapy. The relationship between the stage of cachexia and chemotherapy outcomes is shown in Table 5. There was no statistically significant relationship between the radiological response to chemotherapy (*r* = − 0.366, *p* = 0.179) and the number of chemotherapy cycles delayed (*r* = 0.168, *p* = 0.516) or dose reduced (*r* = 0.161, *p* = 0.543) with cachexia stage; however, there was a moderate negative correlation between the number of chemotherapy cycles completed and cachexia stage (*r* = 0.431, *p* = 0.030) indicating that as the stage of cachexia progressed, the number of chemotherapy cycles completed reduced.

**Table 5:** Relationship between cachexia stage and chemotherapy outcomes

	NC (*n*)	Cachexia (*n*)	RC (*n*)	*r* (*p*)	95% CI
Radiological response to chemotherapy				− 0.366 (0.179)	− 0.390–0.734
Responder	4	3	3		
Non-responder	0	1	2		
Chemotherapy cycles completed				− 0.431 (0.030)^†^	− 0.718 to − 0.053
0	0	3	5		
1	0	0	2		
2	0	1	0		
3	0	0	1		
4	4	3	2		
Chemotherapy cycles delayed				0.168 (0.516)	− 0.346–0.667
0	3	2	2		
1	0	1	2		
2	1	1	1		
Chemotherapy dose reductions				0.161 (0.543)	− 0.415–0.643
0	3	2	3		
1	1	2	1		
2	0	0	1		

^†^Statistically significant *p* values < 0.05

### Sarcopenia, CRP and chemotherapy outcomes

The results did not identify any statistically significant relationships between the presence of sarcopenia and any of the chemotherapy outcomes (Table 6).

**Table 6:** Correlation between the presence of sarcopenia and CRP with chemotherapy outcomes

	CRP			Sarcopenia		
	*r*	*p*	95% CI	*r*	*p*	95% CI
Radiological response	0.524	0.098	0.198*–*0.867	− 0.048	0.886	− 0.764–0.513
Chemotherapy cycles completed	0.466	0.044^†^	− 0.830–0.007	0.091	0.778	− 0.500–0.764
Chemotherapy cycles delayed	0.081	0.813	− 0.596–0.773	0.165	0.605	− 0.499–0.629
Chemotherapy cycles dose reduced	0.616	0.044^†^	0.061–0924	0.429	0.199	0.167–0.816

^†^Statistically significant *p* values < 0.05

CRP was dichotomised for the purpose of this analysis into the upper and lower 50th centiles, and associations with chemotherapy outcomes are shown in Table 7 with significant differences identified between those with a CRP in the lowest 50th centile and the highest 50th centile in relation to chemotherapy dose reductions (*p* = 0.047). Additionally, correlation analysis with CRP (as a continuous variable) and chemotherapy outcomes is shown in Table 6. A moderate negative relationship was identified between CRP and the number of chemotherapy cycles completed (*r* = 0.466, *p* = 0.044) indicating as CRP increased the number of chemotherapy cycles completed reduced. There was also a strong positive relationship between CRP and the number of chemotherapy dose reductions (*r* = 0.616, *p* = 0.044) suggesting that as CRP rises so too does the number of dose reductions required. There was no significant relationship with radiological response or chemotherapy cycles delayed.

**Table 7:** Differences between chemotherapy outcomes in those with a low and high CRP

	CRP < 50th centile	CRP > 50th centile	*p*
Responder	7 (64)	2 (18)	0.109
Non-responder	0 (0)	2 (18)	
No chemo	3 (16)	5 (26)	0.055
Chemo	7 (37)	4 (21)	
No chemo dose reductions	6 (55)	1 (9)	0.047^†^
Chemo dose reductions	1 (9)	3 (27)	
No chemo delays	5 (45)	1 (9)	0.450
Chemo delays	2 (18)	3 (27)	

^†^Statistically significant *p* values < 0.05

## Discussion

The aims of this study were to explore the prevalence and severity of CC in a single tumour population of advanced NSCLC patients and to determine its relationship with chemotherapy outcomes. The results indicate a high prevalence of cachexia in this population, but there was insufficient evidence of a relationship between the stage of cachexia and response to chemotherapy; however, the small sample size is a significant limitation in drawing any strong conclusions.

The prevalence of cachexia by stage was 18% with no cachexia, 18% with PC, 14% with cachexia and 50% with RC. Even with the wide confidence intervals, these results are still suggestive of a high prevalence of at least some degree of cachexia in this population and Vigano et al. [4] found similar results to this study with 29% having no cachexia, 14% PC, 24% cachexia and 34% RC. Using the classification model proposed by Vigano et al. [4], it was possible to categorise all patients with a stage of cachexia where complete data sets were available although this does suggest that this model may have limited applications to the clinical setting if the data required is not always routinely available.

There was insufficient evidence of a relationship between the stage of cachexia and response to chemotherapy, which may be because of the small sample size. Only 13/24 (54%) patients recruited went on to receive chemotherapy with 10 (40% of total sample) having a beneficial response to the treatment. Only a small proportion gaining benefit to treatment is not unexpected in a population of advanced NSCLC patients with other studies showing similar response rates to palliative chemotherapy [24]. Although no relationship between the stage of cachexia and response to chemotherapy was identified, there was a trend towards worsening rates of response as cachexia severity increased. There was a 100% response rate in the no cachexia group, 75% in the cachexia group and only 60% in the RC group. However, this is speculative but may be a trend that warrants further exploration in future research. There was a statistically significant relationship between the number of chemotherapy cycles completed and the stage of cachexia (*r* = 0.431, *p* = 0.030) with those having no cachexia receiving all four planned cycles. This may be expected given those with no cachexia would generally have the greatest performance status and fitness [1]; however, significant findings between *p* = 0.01 and *p* = 0.05 should be treated as tentative, pending further analysis with more data given the small sample size.

The results did not identify any statistically significant relationships between the presence of sarcopenia and any of the chemotherapy outcomes. These findings supported by larger study which included 100 NSCLC patients [25], however, are in direct contrast to other findings that have demonstrated a reduced disease response and increased treatment toxicities with the presence of sarcopenia [9, 10] although these were also relatively small studies, both with sample sizes of 55.

Statistically significant negative relationships were identified between CRP and the number of chemotherapy cycles completed (*r* = −0.466, *p* = 0.044) and the number of dose reductions (*r* = 0.616, *p* = 0.044). No literature was identified that had explored similar relationships as most studies in relation to CRP and cachexia have explored an elevated CRP as an independent predictor of survival [26, 27] but did not explore its associations with outcome to chemotherapy. However, it can be hypothesised that given the integral role of an inflammatory response in the pathophysiology of CC [1, 28] that as this response intensifies so too does the degree of cachexia and hence could predict a reduced tolerance and response to chemotherapy. This provides sufficient evidence to at least further explore the predictive ability of CRP in terms of chemotherapy outcomes and its inclusion as part of routine assessment for newly diagnosed NSCLC patients especially considering that CRP as a component of GPS has been proposed as a potential objective diagnostic tool for cancer cachexia [29] and has been shown to be a better prognostic factor than weight loss [30].

This study included a homogenous population of palliative NSCLC unlike many other cachexia studies that have included a mix of cancer types and stages. There was no sampling with the aim of approaching all patients who met the inclusion criteria in a set period of time. For this reason, the results and conclusions drawn could be generalisable to the wider NSCLC although this should be interpreted with caution considering the low number of patients recruited to this single centre study. This study used a multicomponent cachexia classification tool which is based on the universally accepted criteria for CC [1]. However, this tool has had limited validation and involves a complex grading system that may limit its application to clinical practice.

The major limitation to this study is the small sample size limiting the statistical power and precision of the findings. The small sample size also resulted in the need to collapse the PC and cachexia categories which meant the full extent of the classification model and its categories could not be explored.

Careful planning to overcome some of the pitfalls in recruiting to observational studies in advanced cancer patients is required. Less than 50% of the predicted recruitment rate was achieved with only 24 patients included. This was due to competition with several other observational and intervention studies taking place and because fewer number of patients receiving first line palliative chemotherapy than predicted, instead receiving targeted biological therapies. Although a prospective study, there was still a significant amount of missing data, in particular a lack of availability of blood tests and CT scans which suggests, although important to limit the burden on participants where necessary, additional investigations should be sought with the patient’s consent, to ensure complete data for analysis. Observational studies in advanced lung cancer patients should ideally involve multiple research sites to broaden the access to patients and improve the generalisability of the results. If adequate numbers of patients can be accessed for similar research, the findings here indicate a high uptake to the study can be achieved.

## Conclusions

Limited conclusions can be drawn in terms of clinical practice given the small sample size and limited statistical power of the analysis undertaken. However, the results are suggestive of a high prevalence of cachexia in advanced stage NSCLC patients with a majority presenting with some degree of cachexia at diagnosis. The cachexia assessment tool was able to categorise all patients with complete data sets into an appropriate stage of cachexia; however, this study was not able to provide any validation in terms of how the different stages of cachexia predicted outcome to chemotherapy. A relationship was identified between the increasing severity of cachexia and a lower number of chemotherapy cycles completed, but this needs to be confirmed by larger studies.

Individual variables such as the presence of sarcopenia and CRP appeared to have no associations with chemotherapy outcomes. Although a negative relationship was identified between an increasing CRP and the number of chemotherapy cycles completed and the number of dose reductions required, these need further exploration in larger studies before any firm conclusions can be drawn.

## References

[1] Fearon K, Strasser F, Anker SD, Bosaeus I, Bruera E, Fainsinger RL, Jatoi A, Loprinzi C, MacDonald N, Mantovani G, Davis M, Muscaritoli M, Ottery F, Radbruch L, Ravasco P, Walsh D, Wilcock A, Kaasa S, Baracos VE (2011). Definition and classification of cancer cachexia: an international consensus. Lancet Oncol.

[2] Penet M-F, Bhujwalla Z (2015). Cancer cachexia, recent advances, and future directions. Cancer J.

[3] Sadeghi M, Keshavarz-Fathi M, Baracos V, Arends J, Mahmoudi M, Rezaei N (2018). Cancer cachexia: diagnosis, assessment, and treatment. Crit Rev Oncol Hematol.

[4] Vigano A, Del Fabbro E, Bruera E, Borod M (2012). The cachexia clinic: from staging to managing nutritional and functional problems in advanced cancer patients. Crit Rev Oncog.

[5] Wallengren O, Lundholm K, Bosaeus I (2013). Diagnostic criteria of cancer cachexia: relation to quality of life, exercise capacity and survival in unselected palliative care patients. Support Care Cancer.

[6] Thoresen L, Frykholm G, Lydersen S, Ulveland H, Baracos V, Prado C, Birdsell L, Falkmer U (2013). Nutritional status, cachexia and survival in patients with advanced colorectal carcinoma. Different assessment criteria for nutritional status provide unequal results. Clin Nutr.

[7] Blum D, Stene GB, Solheim TS, Fayers P, Hjermstad MJ, Baracos VE, Fearon K, Strasser F, Kaasa S (2014). Validation of the consensus-definition for cancer cachexia and evaluation of a classification model – a study based on data from an international multicentre project (EPCRC-CSA). Ann Oncol.

[8] Kimura M, Naito T, Kenmotsu H, Taira T, Wakuda K, Oyakawa T, Hisamatsu Y, Tokito T, Imai H, Akamatsu H, Ono A, Kaira K, Murakami H, Endo M, Mori K, Takahashi T, Yamamoto N (2015). Prognostic impact of cancer cachexia in patients with advanced non-small cell lung cancer. Support Care Cancer.

[9] Prado CM, Baracos VE, McCargar LJ, Reiman T, Mourtzakis M, Tonkin K, Mackey JR, Koski S, Pituskin E, Sawyer MB (2009). Sarcopenia as a determinant of chemotherapy toxicity and time to tumor progression in metastatic breast cancer patients receiving capecitabine treatment. Clin Cancer Res.

[10] Antoun S, Baracos VE, Birdsell L, Escudier B, Sawyer MB (2010). Low body mass index and sarcopenia associated with dose-limiting toxicity of sorafenib in patients with renal cell carcinoma. Ann Oncol.

[11] Barret M, Antoun S, Dalban C, Malka D, Mansourbakht T, Zaanan A, Latko E, Taieb J (2014). Sarcopenia is linked to treatment toxicity in patients with metastatic colorectal cancer. Nutr Cancer.

[12] Tan C, Read J, Phan V, Beale P, Peat J, Clarke S (2015). The relationship between nutritional status, inflammatory markers and survival in patients with advanced cancer: a prospective cohort study. Support Care Cancer.

[13] Gioulbasanis I, Georgoulias P, Vlachostergios PJ, Baracos V, Ghosh S, Giannousi Z, Papandreou CN, Mavroudis D, Georgoulias V (2011). Mini Nutritional Assessment (MNA) and biochemical markers of cachexia in metastatic lung cancer patients: interrelations and associations with prognosis. Lung Cancer.

[14] Oken M, Creech R, Tormey D, Horton J, Davis T, McFadden E, Carbone P (1982). Toxicity and response criteria of the Eastern Cooperative Oncology Group. Am J Clin Oncol.

[15] Fess E, Casanova J (1992). Hand grip. Clinical assessment recommendations.

[16] Bohannon RW, Peolsson A, Massy-Westropp N, Desrosiers J, Bear-Lehman J (2006). Reference values for adult grip strength measured with a Jamar dynamometer: a descriptive meta-analysis. Physiotherapy.

[17] WHO (1998). Obesity - preventing and managing the global epidemic.

[18] Baracos V, Reiman T, Mourtzakis M, Gioulbasanis I, Antoun S (2010). Body composition in patients with non–small cell lung cancer: a contemporary view of cancer cachexia with the use of computed tomography image analysis. Am J Clin Nutr.

[19] Baumgartner RN, Koehler KM, Gallagher D, Romero L, Heymsfield SB, Ross RR, Garry PJ, Lindeman RD (1998). Epidemiology of sarcopenia among the elderly in New Mexico. Am J Epidemiol.

[20] Mourtzakis M, Prado C, Lieffers J, Reiman T, McCargar L, Baracos V (2008). A practical and precise approach to quantification of body composition in cancer patients using computed tomography images acquired during routine care. Appl Physiol Nutr Metab.

[21] Moro C, Brunelli C, Miccinesi G, Fallai M, Morino P, Piazza M, Labianca R, Ripamonti C (2006). Edmonton Symptom Assessment Scale: Italian validation in two palliative care settings. Support Care Cancer.

[22] Vigano A, Morais J, Ciutto L, Rosenthall L, di Tomasso J, Khan S, Olders H, Borod M, Kilgour R (2017). Use of routinely available clinical, nutritional, and functional criteria to classify cachexia in advanced cancer patients. Clin Nutr.

[23] Eisenhauer EA, Therasse P, Bogaerts J, Schwartz LH, Sargent D, Ford R, Dancey J, Arbuck S, Gwyther S, Mooney M, Rubinstein L, Shankar L, Dodd L, Kaplan R, Lacombe D, Verweij J (2009). New response evaluation criteria in solid tumours: revised RECIST guideline (version 1.1). Eur J Cancer.

[24] Murphy RA, Mourtzakis M, Chu QSC, Baracos VE, Reiman T, Mazurak VC (2011). Supplementation with fish oil increases first-line chemotherapy efficacy in patients with advanced nonsmall cell lung cancer. Cancer.

[25] Srdic D, Plestina S, Sverko-Peternac A, Nikolac N, Simundic A, Samarzija M (2016). Cancer cachexia, sarcopenia and biochemical markers in patients with advanced non-small cell lung cancer – chemotherapy toxicity and prognostic value. Support Care Cancer.

[26] Brown DJF, Milroy R, Preston T, Mcmillan DC (2007). The relationship between an inflammation-based prognostic score (Glasgow Prognostic Score) and changes in serum biochemical variables in patients with advanced lung and gastrointestinal cancer, J. Clin Pathol.

[27] Pastore CA, Orlandi SP, Gonzalez MC (2013). Association between an inflammatory-nutritional index and nutritional status in cancer patients. Nutr Hosp.

[28] Skipworth RJE, Stewart GD, Dejong CHC, Preston T, Fearon K (2007). Pathophysiology of cancer cachexia: much more than host–tumour interaction?. Clin Nutr.

[29] Douglas E, McMillan D (2014). Towards a simple objective framework for the investigation and treatment of cancer cachexia: the Glasgow Prognostic Score. Cancer Treat Rev.

[30] Simmons C, Koinis F, Fallon M, Fearon K, Bowden J, Solheim T, Gronberg B, McMillan D, Gioulbasanis LJ (2015). Prognosis in advanced lung cancer - a prospective study examining key clinical pathological factors. Lung Cancer.

